# α-Cyclodextrins Polyrotaxane Loading Silver Sulfadiazine

**DOI:** 10.3390/polym10020190

**Published:** 2018-02-14

**Authors:** Sa Liu, Chunting Zhong, Weiwei Wang, Yongguang Jia, Lin Wang, Li Ren

**Affiliations:** 1 School of Materials Science and Engineering, South China University of Technology, Guangzhou 510641, China; sliu@scut.edu.cn (S.L.); msctzhong@mail.scut.edu.cn (C.Z.); wei880719@163.com (W.W.); ygjia@scut.edu.cn (Y.J.); wanglin3@scut.edu.cn (L.W.); 2 National Engineering Research Center for Tissue Restoration and Reconstruction, Guangzhou 510006, China

**Keywords:** polyrotaxane, silver sulfadiazine, mechanism, antibacterial

## Abstract

As a drug carrier, polyrotaxane (PR) has been used for targeted delivery and sustained release of drugs, whereas silver sulfadiazine (SD-Ag) is an emerging antibiotic agent. PR was synthesized by the use of α-cyclodextrin (CD) and poly(ethylene glycol) (PEG), and a specific antibacterial material (PR-(SD-Ag)) was then prepared by loading SD-Ag onto PR with different mass ratios. The loading capacity and the encapsulation efficiency were 90% at a mass ratio of 1:1 of PR and SD-Ag. SD-Ag was released stably and slowly within 6 d in vitro, and its cumulative release reached more than 85%. The mechanism of PR loading SD-Ag might be that SD-Ag attached to the edge of α-CD through hydrogen bonding. PR-(SD-Ag) showed a higher light stability than SD-Ag and held excellent antibacterial properties against *Escherichia coli* (*E. coli*) and *Staphylococcus aureus* (*S. aureus*).

## 1. Introduction

To improve the health of the population, a variety of antimicrobial agents have been used to suppress and kill harmful microorganisms in people’s living environments [1,2,3]. After the discovery and application of antibiotics, antibacterial and bactericidal properties seem increasingly prominent. However, the emergence of super-resistant strains of bacteria threatens human health, resulting in the extensive usage of antibiotics [4]. The research on broad-spectrum antibacterial materials showing nontoxicity and bio-heat resistance has become extremely urgent [5,6,7].

Compared to other kinds of antibacterial agents, silver-based antimicrobial materials are effective and safe. They have been widely used and have become a focus of commercialization in antimicrobial research [8,9]. For example, silver sulfadiazine (SD-Ag) has received wide-spread acceptance as a topical agent to control bacterial infections [10]. The key of bacteriostatic agents is silver ions [11], which significantly affect the treatment of burn wounds and the promotion of wound healing and infection control. However, crystalline silver sulfadiazine easily deteriorates under the influence of light or heat. It can dissolve in acid or ammonia aqueous media, but not in ethanol, chloroform, and some other organic solvents. These drawbacks limit the application of these silver-based antimicrobial agents.

Polyrotaxane (PR) has a necklace-like molecular structure, where many cyclic molecules are threaded by a linear molecule ending in two bulky groups to avoid the dissociation of the cyclic molecules [12]. Recently, PR has attracted a great deal of interest in the production of various raw materials [13,14,15] due to its specific characteristics of free sliding and/or rotating threaded cyclic molecules. Moreover, PR can be easily metabolized by the body to reduce cytotoxicity [16,17,18]. As a drug carrier, PR has been used for targeted drug-delivery and the sustained release of drugs, such as peptides [19,20,21] and genes [22,23,24]. Circumstantially, this strategy can improve the biological properties of drugs, such as low haemolysis, lasting pharmacodynamics, biodegradable properties, and low cytotoxicity. Thus, these PRs could be excellent carriers for SD-Ag-based antibacterial materials.

In this paper, the antibacterial agent SD-Ag was loaded onto PR and antibacterial complexes PR-(SD-Ag), and different mass ratios of PR/SD-Ag were prepared. The drug loading content, the in vitro drug release, light stability, and loading mechanism are discussed. Antibacterial properties of PR-(SD-Ag) against *Escherichia coli* (*E. coli*) and *Staphylococcus aureus* (*S. aureus*) were also tested.

## 2. Materials and Methods

### 2.1. Materials

α-Cyclodextrins (α-CDs) were purchased from Shandong BinzhouZhiyuan Bio-Technology Co., Ltd. (Binzhou, China) (α-CD content > 99%); poly(ethylene glycol) (PEG 35,000 and PEG 20,000) was purchased from Sigma (Shanghai, China). The free-base forms of 1-adamantanamine and silver sulfadiazine (SD-Ag) were purchased from the Aladdin Reagent Company (Shanghai, China). All reagents were of experimental grade and were used without further purification. All solvents were dehydrated.

### 2.2. Synthesis of PR

PEG (10 g) was oxidized using 2,2,6,6-Tetramethylpiperidine 1-Oxyl (TEMPO) (100 mg), NaBr (100 mg), and aqueous NaClO (10 mL, available chlorine > 5.0%) in water (100 mL). After stirring at room temperature (pH 10–11) for 15 min, the oxidation reaction was quenched by adding ethanol (10 mL). Then, HCl solution was used to lower the pH to less than 2, and the solution was extracted three times using CH_2_Cl_2_. The organic phase was dried in vacuo. The crude residue was dissolved in hot ethanol (250 mL) and precipitated at −20 °C overnight. PEG-COOH was obtained after the second recrystallization with ethanol. After titration with 0.01 mol·L^−1^ NaOH and a percent conversion of >99%, it was indicated that PEG (20,000 and 35,000) were converted to the corresponding PEG-COOH.

PEG-COOH (1.5 g) and α-CD (6 g) were dissolved in water (100 mL). After stirring at 70 °C for 20 h and 4 °C for 48 h in succession, a white precipitate appeared. After freeze-drying, the pseudo-polyrotaxane was mixed with adamantanamine (0.8 g), (benzotriazol1-yloxy) tris-(dimethylamino) phosphonium hexafluorophosphate (0.24 g), and ethyldiisopropylamine (EDIPA) (0.1 mL), and was then dissolved in dehydrated dimethylformamide (DMF) (100 mL). The slurry-like mixture was stirred at room temperature overnight, then washed with DMF/methanol (1:1) and methanol in succession. The PR solid was obtained after the white residue was dissolved in dimethyl sulfoxide (DMSO) (40 mL), precipitated, and washed with water (400 mL), and, finally, freeze-dried. PR1 t and PR2 t (based on molecular weight 20,000 and 35,000) were prepared. ^1^H NMR (400 MHz, DMSO-d_6_) δ (ppm) 5.63 (s, 1H, O_2_–H), 5.48 (s, 1H, O_3_–H), 4.90 (s, 1H, C_1_–H), 4.44 (s, 1H, O_6_–H), 3.69 (d, *J* = 32.4 Hz, 3H, C_3,5,6_–H), 3.51 (s, 5H, C_PEG_-H), 2.01 (s, 1H, N–H), 1.94 (s, 1H, N–H), 1.62 (s, 1H, N–H).

### 2.3. Preparation of PR-(SD-Ag)

According to the mass ratios 1:1, 1.5:1, and 2:1 of PR and SD-Ag, the different mixtures were each dissolved in DMSO and protected from light. After stirring for 6 h at room temperature and dialyzing in deionized water for 4 d at 25 °C, the liquid was freeze-dried, generating white solid PR-(SD-Ag). ^1^H NMR (400 MHz, DMSO-d_6_) δ (ppm) 8.38 (d, *J* = 4.9 Hz, 1H, P_1_–H), 7.62 (d, *J* = 8.6 Hz, 1H, B_1_–H), 6.77 (s, 1H, B_2_–H), 6.51 (d, *J* = 8.6 Hz, 1H, P_2_–H), 5.68 (s, 1H, O_2_–H), 5.49 (s, 1H, O_3_–H), 4.80 (s, 1H, C_1_–H), 4.45 (s, 1H, O_6_–H), 3.69 (d, *J* = 37.5 Hz, 3H, C_3,5,6_–H), 3.51 (s, 3H, C_PEG_–H), 3.28 (s, 2H, C_2,4_–H).

### 2.4. Fourier Transform Infrared (FTIR) Spectroscopy

Absorbance spectra were recorded using the KBr pellet method with a VECTOR-22 FTIR spectrometer (Bruker, Ettlingen, Germany). The spectra were collected in the region of 4000 to 400 cm^−1^.

### 2.5. X-ray Diffraction

X-ray diffraction was recorded on powdered samples using a D8 ADVANCE X-ray diffractometer (Rigaku, Tokyo, Japan). The conditions were set to the following: 40 kV (voltage), 40 mA (current), and 1.54 A˚ (wavelength). The samples were mounted on a circular sample holder and then sealed with Scotch tape. The proportional counter detector collected data at a rate of 2θ = 10°·min^−1^ over the following range: 2θ = 5–90°.

### 2.6. ^1^H NMR Spectroscopy

^1^H NMR spectra were recorded on a Bruker AVANCE 400 MHz spectrometer (Bruker, Ettlingen, Germany) at room temperature, using DMSO-d6 as solvent and tetramethylsilane (TMS) as internal standard.

### 2.7. Light Stability

Different PR-(SD-Ag) systems were dissolved in DMSO. Then, the solutions were placed under fluorescent light for an irradiation period, after which a change in color could be observed. The solution concentration was 0.02 g/mL.

### 2.8. Analysis of Drug Content

The sample was placed in a small beaker; then, a small amount of concentrated nitric acid was added for dissolution. After pouring into a 50 mL volumetric flask, phosphate-buffered saline (PBS) solution was added as a diluent. The absorbance values were obtained using a UV/visible spectrophotometer (Persee, Beijing, China) at 245 nm. All the experiments were done in triplicate.

### 2.9. In Vitro Release Studies

Quantitative PR-(SD-Ag) was placed in a dialysis bag immersed in phosphate-buffered saline solution (PBS, 50 mL, pH 7.4), and drug release was performed at 37 °C with stirring. Supernatants were removed at every desired time interval to determine the amount of SD-Ag released from PR, which was then analyzed using a UV/visible spectrophotometer at 245 nm. Then, the supernatant was added again to maintain a constant volume. The amount of SD-Ag was previously quantified using a built analytical curve, which was designed using the absorbance versus standard SD-Ag solution, varying from 2.5 to 20 μg·mL^−1^. The linear correlation coefficient (*R*^2^) was 0.9988.

## 3. Results and Discussion

### 3.1. Preparation of the PR-(SD-Ag) Complex 

FTIR spectra of SD-Ag, PR, and their complex PR-(SD-Ag), as well as the physical mixture in the region from 500 to 4000 cm^−1^, are presented in Figure S1. In the spectrum of SD-Ag, peaks at 3394, 3344, and 3261 cm^−1^ were attributed to N–H stretching vibrations. Peaks at 1654, 1593, and 1583 cm^−1^ were attributed to the phenyl and pyrimidinyl rings. Asymmetric and symmetric vibrations of γ (SO_2_) appear at 1354 and 1137 cm^−1^, respectively. This is in good agreement with literature data. In the spectrum of pure PR, the broad band at 3356 cm^−1^ and the weak band at 1643 cm^−1^ were assigned to the stretching vibration of the hydroxyl groups in α-CD and the hydration of water, respectively, which is similar to that of pseudo-PR between PEG and α-CD (supporting information) [25]. The spectrum of PR-(SD-Ag) contains the characteristic absorption peaks of PR, but lost some of SD-Ag. A new peak appeared at 1586 cm^−1^, slightly higher than 1583 cm^−1^ in the SD-Ag spectrum, which is related to the unusual interactions around the phenyl and pyrimidinyl rings. The IR spectrum of the physical mixture of PR and SD-Ag clearly showed almost all the characteristic absorption peaks. These results indicate that PR-(SD-Ag) is not a simple mixture of PR and SD-Ag.

PR, SD-Ag, and PR-(SD-Ag) were characterized using XRD (Figure S2). There was a broad diffraction peak at 2θ 11.10°, a distinct peak at 2θ 19.84°, and a relatively insignificant peak at 2θ 13.32° for PR. The peaks of SD-Ag were more complicated, and were mainly at 2θ 8.80°, 10.21°, and 18.49°. Compared to SD-Ag and PR, the peaks of PR-(SD-Ag) moved slightly, appearing at 2θ 8.86°, 10.38°, and 19.91°, respectively, and a new peak was observed at 2θ 11.46°. This may suggest that there is a specific interaction between SD-Ag and α-CD in PR.

**Figure 1:**
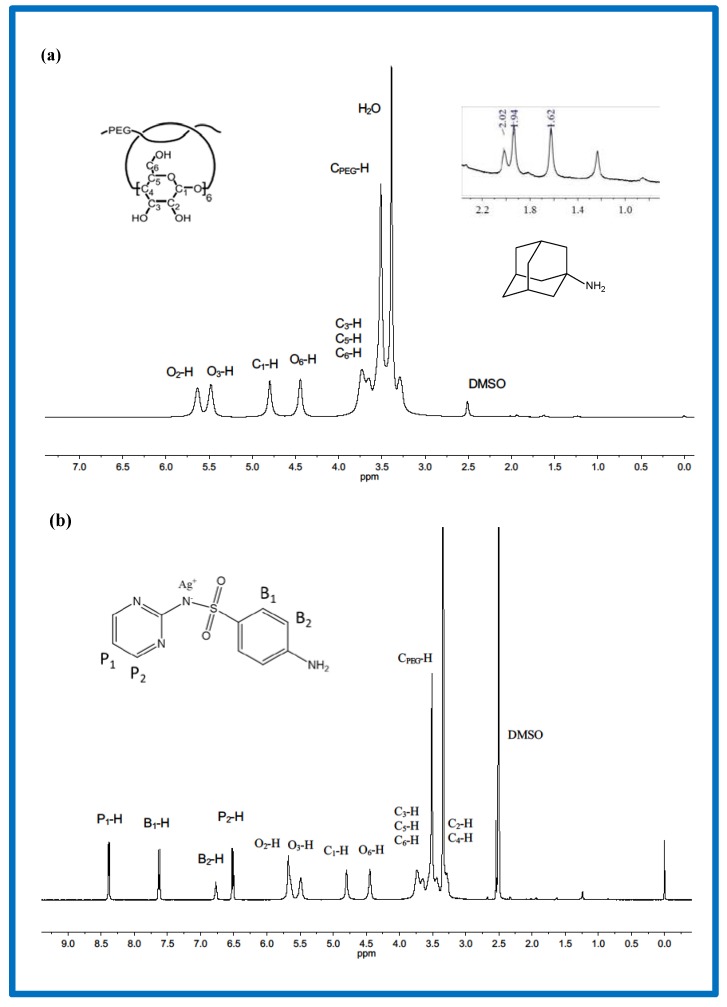
^1^H NMR spectra of (**a**) polyrotaxane (PR) and (**b**) polyrotaxane-silver sulfadiazine complex (PR-(SD-Ag)) in DMSO-d_6_.

PRs were then systematically characterized using ^1^H NMR analysis (Figure 1a). All peaks of the protons on PEG and α-CD were confirmed. The signal peaks of PEG methylene were around 3.51 ppm. The chemical shifts of protons on α-CD appeared at 5.63 ppm (O_2_–H), 5.48 ppm (O_3_–H), 4.44 ppm (O_6_–H), and 4.80 (C_1_–H), 3.80–3.20 ppm (C_2,3,4,5,6_-H). Additionally, peaks at 2.02, 1.94, and 1.62 ppm showed the protons of the carbon skeleton in amantadine. This manifested the formation of PR after the end-capping reaction between amino groups in amantadine and terminal carboxyl groups in PEG. ^1^H NMR analysis was used to confirm the structure of PR-(SD-Ag) (Figure 1b). The characteristic peaks at 5.68 ppm (O_2_–H), 5.49 ppm (O_3_–H), and 4.45 ppm (O_6_–H) express the information of hydroxyl in α-CD. Peaks at 4.80 and 3.20–3.80 ppm showed the information of CH in α-CD, and the peak at 3.51 ppm was attributed to the methylene of PEG. The new peaks around 6.50–8.39 ppm were attributed to the benzene rings and pyrimidine rings in SD-Ag. However, the signals from the amino groups in SD-Ag disappeared and the chemical shifts of the hydroxyl group in α-CD of PR-(SD-Ag) shifted downward slightly compared with that of PR. These results also indicated that the complex formed upon mixing PR and SD-Ag.

### 3.2. Drug Loading and In Vitro Release

**Figure 2:**
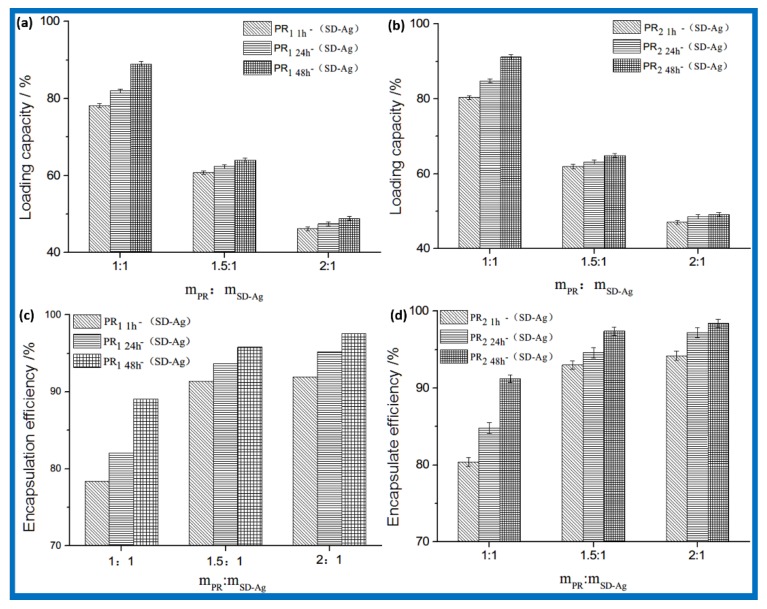
The drug loading capacity of (**a**) PR_1 t_-(SD-Ag), (**b**) PR_2 t_-(SD-Ag), and the encapsulation efficiency of (**c**) PR_1 t_-(SD-Ag), (**d**) PR_2 t_-(SD-Ag).

Strategies were used through designing different mass ratios of PR/SD-Ag to study the drug loading and encapsulation efficiency of PR_1 t_-(SD-Ag) and PR_2 t_-(SD-Ag) (Figure 2). They presented similar variational tendencies in these histograms. Maintaining a certain mass ratio of PR/SD-Ag, the increased content of α-CD in PR led to an increase in drug loading capacity and encapsulation rates. Maintaining a certain content of α-CD, the higher the mass ratio of PR/SD-Ag, the lower the drug loading capacity, while the encapsulation efficiency maintained a sustainable growth with an increased quantity ratio. As both the drug loading capacity and the encapsulation efficiency were higher than 78.2%, a 1:1 mass ratio of PR/SD-Ag is desired. The greatest drug loading capacity was 89.0% for PR_1 t_-(SD-Ag) and 91.2% for PR_2 t_-(SD-Ag). The encapsulation rates were 78.2%–90% for PR_1 t_-(SD-Ag) and 80.4%–98.4% for PR_2 t_-(SD-Ag). PR_2 t_-(SD-Ag) showed higher drug loading capacity and encapsulation rates than PR_1 t_-(SD-Ag). This could be attributed to the PEG chain length. The longer the PEG chain, the more the α-CD are bunched, which causes more silver sulfadiazine to be encapsulated. In summary, a 1:1 mass ratio of PR/SD-Ag was optimal, and more SD-Ag was encapsulated using a smaller amount of PR.

**Figure 3:**
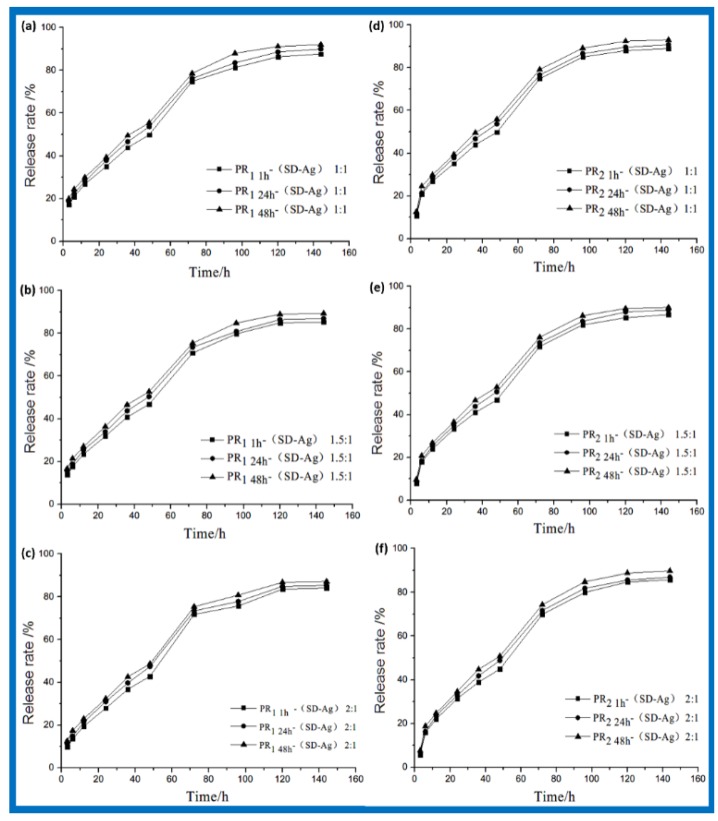
In vitro release profile of (**a**—**c**) PR_1 t_-(SD-Ag) and (**d**—**f**) PR_2 t_-(SD-Ag) with different mass ratios of polyrotaxane and silver sulfadiazine.

Figure 3 shows the process of drug release for PR_1 t_-(SD-Ag) and PR_2 t_-(SD-Ag) in different mass ratios of PR/SD-Ag in a PBS solution (pH at 7.4) at 37 °C. All samples exhibited a similar release tendency. At an earlier stage, the accumulating release rates increased slowly and linearly at a 1:1 mass ratio under vibration. However, the linear regularities became unclear at 1.5:1 and 2:1 mass ratios. After 72 h, the accumulation release rates slowed down gradually until reaching the maximum value, which was about 88% for PR_1 t_-(SD-Ag) and 93% forPR_2 t_-(SD-Ag). Circumstantially, the accumulating release rates of PR_1 1 h_-(SD-Ag), PR_1 24 h_-(SD-Ag), and PR_1 48 h_-(SD-Ag) increased successively (Figure 3a–c). Maintaining a certain time, the relationships for different mass ratios were PR_1 t_-(SD-Ag) 1:1 > PR_1 t_-(SD-Ag) 1.5:1 > PR_1 t_-(SD-Ag) 2:1. These phenomena were attributed to the change in viscosity. The higher the amount of PR, the higher the viscosity of the solution. In proportion, the free volume in the system decreased, which restricted the movement of α-CD. The relation of PR_2 t_-(SD-Ag) was consistent with that of PR_1 t_-(SD-Ag). In summary, PR-(SD-Ag) was expected to exhibit a slow and steady upper release and a high release ratio without an initial burst release.

### 3.3. Antibacterial Studies and Light Stability

**Figure 4:**
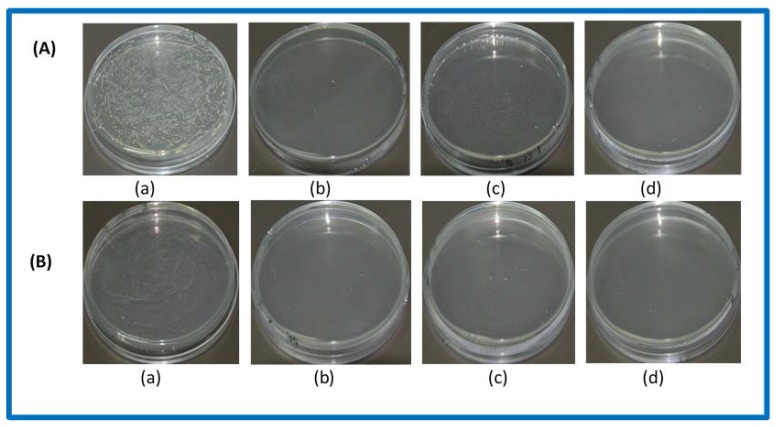
The antibacterial effect of PR-(SD-Ag) on (**A**) *E. coli* and (**B**) *S. aureus*: (**a**) blank groups (PR_2 24 h_); (**b**) PR_2 24 h_-(SD-Ag)2:1; (**c**) PR_2 24 h_-(SD-Ag)1.5:1; (**d**) PR_2 24 h_-(SD-Ag)1:1.

Figure 4 shows the antibacterial effects of PR-(SD-Ag) on *E. coli* and *S. aureus* (1 × 10^7^ CFUmL^−1^), respectively. Compared with the blank group’s PR (Figure 4A(a),B(a)), all experimental groups expressed good antibacterial effects. The four-hour antibacterial rate of PR_1 t_-(SD-Ag) and PR_2 t_-(SD-Ag) for *E. coli* and *S. aureus* were tested, and the results showed that the antibacterial rates of all groups were higher than 98%. It was found that the antibacterial effects of PR-(SD-Ag) with different mass ratios of polyrotaxane and silver sulfadiazine were slightly different. When the mass ratio was 1:1, the antibacterial effect of PR-(SD-Ag) was the best. The maximum antibacterial rate reached 99.9% for *E. coli* and 99.6% for *S. aureus*, respectively. This is consistent with the results of in vitro drug release for PR-(SD-Ag).

**Figure 5:**
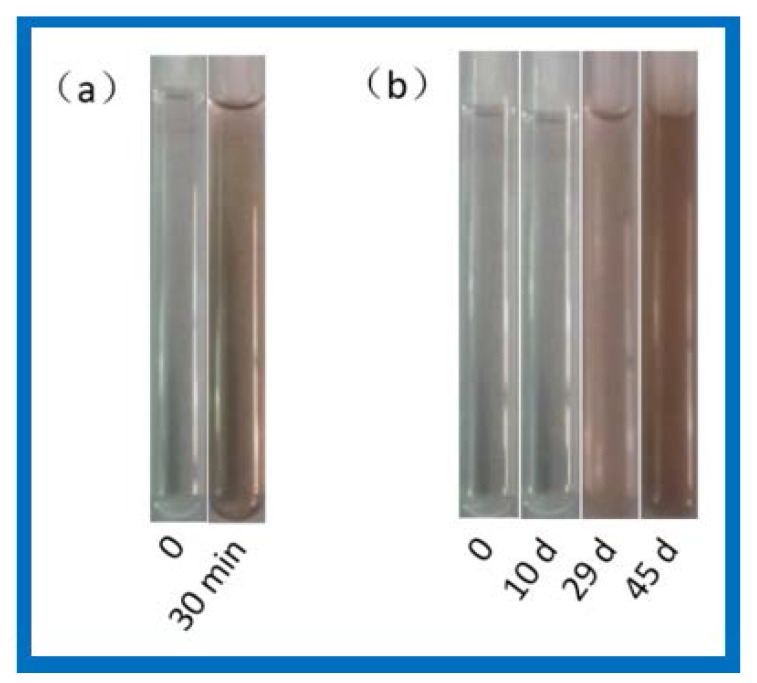
Comparison of light stability: (**a**) The color of SD-Ag solution became brown after being exposed to fluorescent light for 30 min; (**b**) the color of PR-(SD-Ag) did not change until day 29, when it became slightly transparent and red, and dark brown after day 45.

Light stability of SD-Ag and PR-(SD-Ag) was compared under fluorescent light for different amounts of time (Figure 5). Except for manually-added light conditions, experiments proceeded at room temperature, and were acquiescently protected from light. PR-(SD-Ag) and SD-Ag were each dissolved in a small amount of ammonia water. Then, they were respectively diluted with DMSO. The color of the SD-Ag solution became brown after being exposed to fluorescent light for 30 min (Figure 5a). After being exposed to fluorescent lamp irradiation for 10 d, the color of PR-(SD-Ag) did not change (Figure 5b). After 29 d, it became slightly transparent and red, and turned dark brown after 45 d. These results indicate that the light stability of SD-Ag in a complex improved greatly, perhaps due to the formation of an inclusion with PR.

### 3.4. The Mechanism of PR Loading SD-Ag

**Figure 6:**
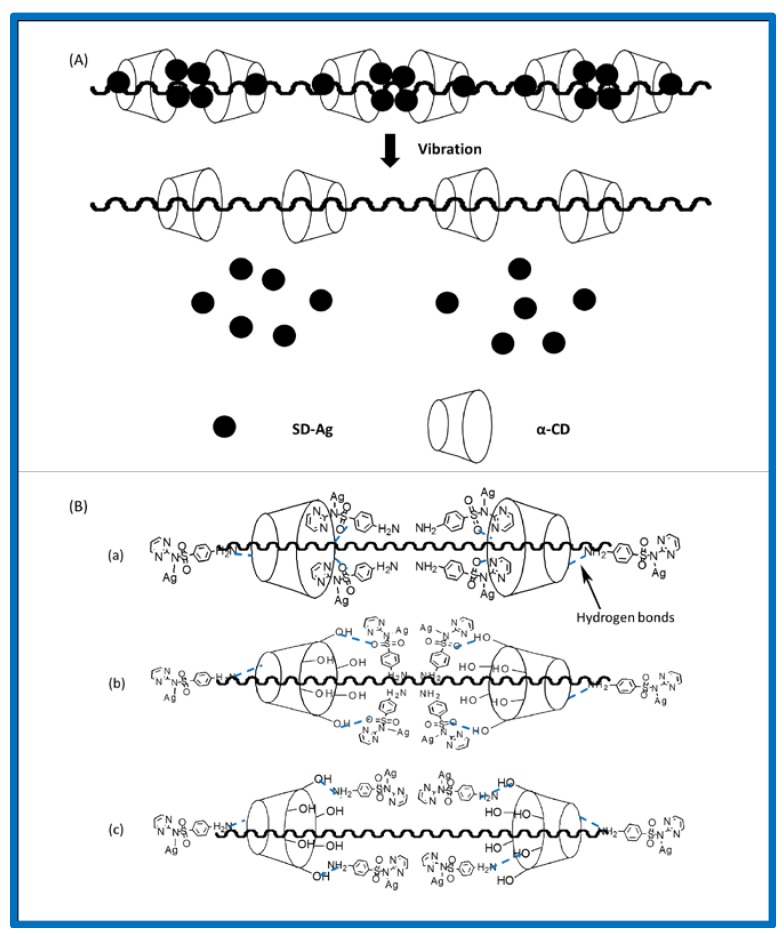
The mechanism of PR loading SD-Ag: (**A**) Drug release model; (**B**) combination through (**a**) N···H···N hydrogen bonds between –NH_2_ in SD-Ag, O···H···O hydrogen bonds between –OH in α-cyclodextrin (CD) and O=S=O in SD-Ag and partial inclusion of pyrimidine rings by α-CD cavity; (**b**) N--H--N hydrogen bonds between –NH_2_ in SD-Ag and O···H···O hydrogen bonds between–OH in α-CD and O=S=O in SD-Ag; (**c**) O···H···N hydrogen bonds between –OH in α-CD and –NH_2_ in SD-Ag.

According to FTIR, XRD, and ^1^H NMR spectroscopic analyses, it was confirmed that PR-(SD-Ag) was not a simple mixture of PR and SD-Ag. Numerous studies have clarified the size-matching law in PR formation [25]. The sizes of α-CD cavities (4.70–5.30 Å) and SD-Ag (4.20–4.70 Å) match [26]. However, it is almost impossible to form complete host–guest inclusion between α-CD and SD-Ag due to PEG chains occupying the α-CD cavity. It is probable that complexes were formed by hydrogen bonding between SD-Ag and the α-CD on the PEG. The interactions of protons in pyrimidine and benzene rings with the α-CD cavity protons lead to a downfield shift of δ [26,27]. The above ^1^H NMR information (a small downfield shift of O-H in α-CD, Figure 1) demonstrate the formation of hydrogen bonds between α-CD and SD-Ag. The molar ratios of α-CD and SD-Ag were estimated to be approximately 2:5.4, based on the NMR integration ratio. Accordingly, the model of each of the two α-CD molecules including four-to-six SD-Ag molecules showed a great advantage because of the hydrophobic cavity environment and abundant exocoel hydroxyls in α-CD. Herein, we describe the model of drug release (Figure 6A), as well as the main complex mechanisms (Figure 6B). First, after the aggregation of four SD-Ag molecules through N···H···N hydrogen bonds between –NH_2_, four pyrimidine rings can be partially included by the α-CD cavity [26] under the protection of O···H···O hydrogen bonds between –OH and O=S=O (Figure 6B(a)). This might be the most stable combination. Second, the combination through N···H···N hydrogen bonds between –NH_2_ and O···H···O hydrogen bonds between –OH and O=S=O might also form a stable complex (Figure 6B(b)). Thirdly, the combination of numerous O···H···N hydrogen bonds between –OH and –NH_2_ showed relative stability (Figure 6B(c)). In these cases, more SD-Ag molecules might attach to the small edge of α-CD through O···H···N hydrogen bonds between –OH and –NH_2_. However, non-covalent bonding between PEG and SD-Ag at all these stages—which might facilitate a boost in drug loading—could not be neglected. When releasing SD-Ag, the weak binding patterns might first be disrupted at 37 °C. Due to the balance between the breaking and linking of partial hydrogen bonds, there was no initial burst release (Figure 3). Later, the stable states were disrupted step-by-step, presenting a slow and steady upper release. Under the protection of PR, SD-Ag became more stable, even when exposed to light (Figure 5).

## 4. Conclusions

In this article, two kinds of PR of PEG toward α-CD were synthesized, and the antibacterial agent SD-Ag was loaded into these PRs in a controlled manner. The loading capacity and encapsulation efficiency of the resulting antibacterial materials, PR-(SD-Ag), were both near 90% when the mass ratio of PR/SD-Ag was 1:1. SD-Ag could be stably and slowly released within 6 d in vitro, and showed release rates of over 85%. PR-(SD-Ag) held excellent antibacterial properties against *E. coli* and *S. aureus*. Moreover, PR-(SD-Ag) had a better light stability compared with SD-Ag alone. The method of using PR as an antibacterial drug carrier will provide more choices in the preparation of antibacterial materials. These PR-(SD-Ag) can be used as a potential antibacterial drug in the future.

## Supplementary Materials

The following are available online at http://www.mdpi.com/2073-4360/10/2/190/s1, Figure S1: The FTIR spectra of PR, SD-Ag, PR+SD-Ag and PR-(SD-Ag), Figure S2: The XRD patterns of PR-(SD-Ag), SD-Ag and PR, Table S1: Antibacterial rate of PR_1 t_-(SD-Ag) and PR_2 t_-(SD-Ag) on *E. coli* and *S. aureus*.

## References

[1] Heydari B., Khalili H., Karimzadeh I., Emadi-Kochak H. (2016). Clinical, paraclinical, and antimicrobial resistance features of community-acquired acute bacterial meningitis at a large infectious diseases ward in Tehran, Iran. Iran. J. Pharm. Res..

[2] Niu F.G., Pan W.C., Su Y.J., Yang Y.J. (2016). Physical and antimicrobial properties of thyme oil emulsions stabilized by ovalbumin and gum Arabic. Food Chem..

[3] Przybylski R., Firdaous L., Chataigne G., Dhulster P., Nedjar N. (2016). Production of an antimicrobial peptide derived from slaughterhouse by-product and its potential application on meat as preservative. Food Chem..

[4] Vespa P.M. (2014). Fever in critical neurologic illness. JAMA-J. Am. Med. Assoc..

[5] Stretton S., Gopinathan U., Willcox M.D.P. (2002). Corneal ulceration in pediatric patients. Paediatr. Drugs.

[6] Kong M., Chen X.G., Xing K., Park H.J. (2010). Antimicrobial properties of chitosan and mode of action: A state of the art review. Int. J. Food Microbiol..

[7] Jiang L., Wang F., Han F., Prinyawiwatkul W., No H.K., Ge B. (2013). Evaluation of diffusion and dilution methods to determine the antimicrobial activity of water-soluble chitosan derivatives. J. Appl. Microbiol..

[8] Yang W.M., Fang W.Y. (1998). Antimicrobial plastics. Fine Petrochem..

[9] Rai M., Yadav A., Gad A. (2009). Silver nanoparticles as a new generation of antimicrobials. Biotechnol. Adv..

[10] Fan L.H.F., Zhao J.J., Huang J., Xu Y.M. (2006). Polyelectrolyte sponges with antimicrobial functions based on chitosan and sodium alginate. J. Wuhan Univ. Technol..

[11] Rosenkranz H.S., Carr H.S. (1972). Silver sulfadiazine: Effect on the growth and metabolism of bacteria. Antimicrob. Agents Chemother..

[12] Araki J., Kataoka T., Ito K. (2008). Preparation of a “sliding graft copolymer”, an organic solvent-soluble polyrotaxane containing mobile side chains, and its application for a crosslinked elastomeric supramolecular film. Soft Matter.

[13] Araki J., Ito K. (2007). Recent advances in the preparation of cyclodextrin-based polyrotaxanes and their applications to soft materials. Soft Matter.

[14] Li J., Loh X.J. (2008). Cyclodextrin-based supramolecular architectures: Syntheses, structures, and applications for drug and gene delivery. Adv. Drug Deliv. Rev..

[15] Harada A., Hashidzume A., Yamaguchi H., Takashima Y. (2009). Polymeric rotaxanes. Chem. Rev..

[16] Lin L., Dong M., Liu C., Wei C., Wang Y., Sun H., Ye H. (2014). A supramolecular strategy for self-mobile adsorption sites in affinity membrane. Macromol. Rapid Commun..

[17] Tardy B.L., Dam H.H., Kamphuis M.M., Richardson J.J., Caruso F. (2014). Self-assembled stimuli-responsive polyrotaxane core-shell particles. Biomacromolecules.

[18] Tan S., Nam E., Cui J., Xu C., Fu Q., Ren J.M., Wong E.H.H., Ladewig K., Caruso F., Blencowe A. (2015). Fabrication of ultra-thin polyrotaxane-based films via solid-state continuous assembly of polymers. Chem. Commun..

[19] Moon C., Kwon Y.M., Lee W.K., Park Y.J., Yang V.C. (2007). In vitro assessment of a novel polyrotaxane-based drug delivery system integrated with a cell-penetrating peptide. J. Control. Release.

[20] Zhang J.X., Ma P.X. (2013). Cyclodextrin-based supramolecular systems for drug delivery: Recent progress and future perspective. Adv. Drug Deliv. Rev..

[21] Inoue Y., Ye L., Ishihara K., Yui N. (2012). Preparation and surface properties of polyrotaxane-containing tri-block copolymers as a design for dynamic biomaterials surfaces. Colloids Surf. B.

[22] Ooya T., Choi H.S., Yamashita A., Yui N., Sugaya Y., Kano A., Maruyama A., Akita H., Ito R., Kogure K. (2006). Biocleavable polyrotaxane-plasmid DNA polyplex for enhanced gene delivery. J. Am. Chem. Soc..

[23] Yamashita A., Yui N., Ooya T., Kano A., Maruyama A., Akita H., Kogure K., Harashima H. (2006). Synthesis of a biocleavable polyrotaxane-plasmid DNA (pDNA) polyplex and its use for the rapid nonviral delivery of pDNA to cell nuclei. Nat. Protoc..

[24] Zhang L., Su T., He B., Gu Z. (2014). Self-assembly polyrotaxanes nanoparticles as carriers for anticancer drug methotrexate delivery. Nano-Micro Lett..

[25] Harada A., Li J., Kamachi M. (1993). Preparation and properties of inclusion complexes of Poly(ethy1eneglycol) with α-cyclodextrin. Macromolecules.

[26] Rajendiran N., Venkatesh G., Saravanan J. (2014). Supramolecular aggregates formed by sulfadiazine and sulfisomidine inclusion complexes with α- and β-cyclodextrins. Spectrochim. Acta A.

[27] Rajendiran N., Thulasidhasan J. (2015). Interaction of sulfanilamide and sulfamethoxazole with bovine serum albumin and adenine: Spectroscopic and molecular docking investigations. Spectrochim. Acta A.

