# Supplementary material for: α-Cyclodextrins Polyrotaxane Loading Silver Sulfadiazine
Source: Polymers (Basel). 2018 Feb 14;10(2):190. doi: 10.3390/polym10020190 (PMC6415174; doi:10.3390/polym10020190)
Supplement: Supplementary file 1 [file polymers-10-00190-s001.pdf]

polymers

MDPI

Supplementary Materials

α-Cyclodextrins Polyrotaxane Loading Sliver Sulfadiazine

Sa Liu [1,2], Chunting Zhong [1,2], Weiwei Wang [1,2], Yongguang Jia [1,2], Lin Wang [1,2] and Li Ren [1,2,*]

[1] School of Materials Science and Engineering, South China University of Technology, Guangzhou 510641, China; sliu@scut.edu.cn (S.L.); msctzhong@mail.scut.edu.cn (C.Z.); wei880719@163.com (W.W.); ygjia@scut.edu.cn (Y.J.); wanglin3@scut.edu.cn (L.W.)

[2] National Engineering Research Center for Tissue Restoration and Reconstruction, Guangzhou 510006, China

* Correspondence: psliren@scut.edu.cn; Tel.: +86-20-2223-6528

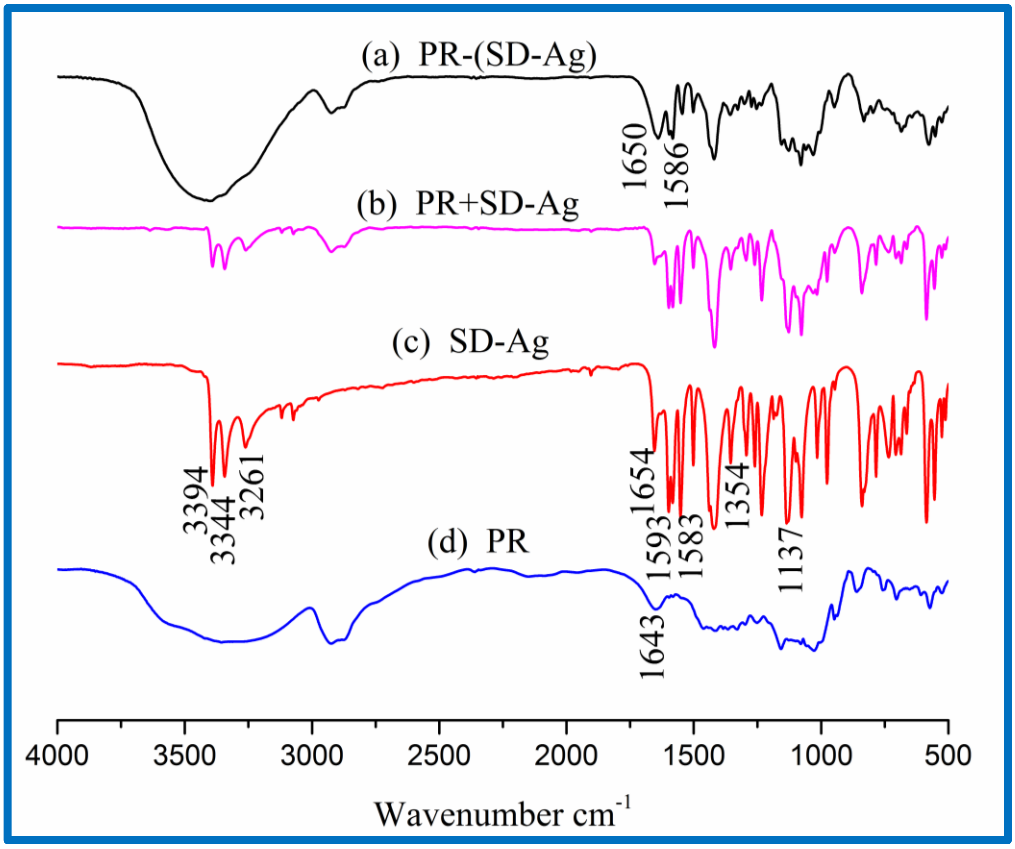

Figure S1. The FTIR spectra of PR, SD-Ag, PR+SD-Ag and PR-(SD-Ag).

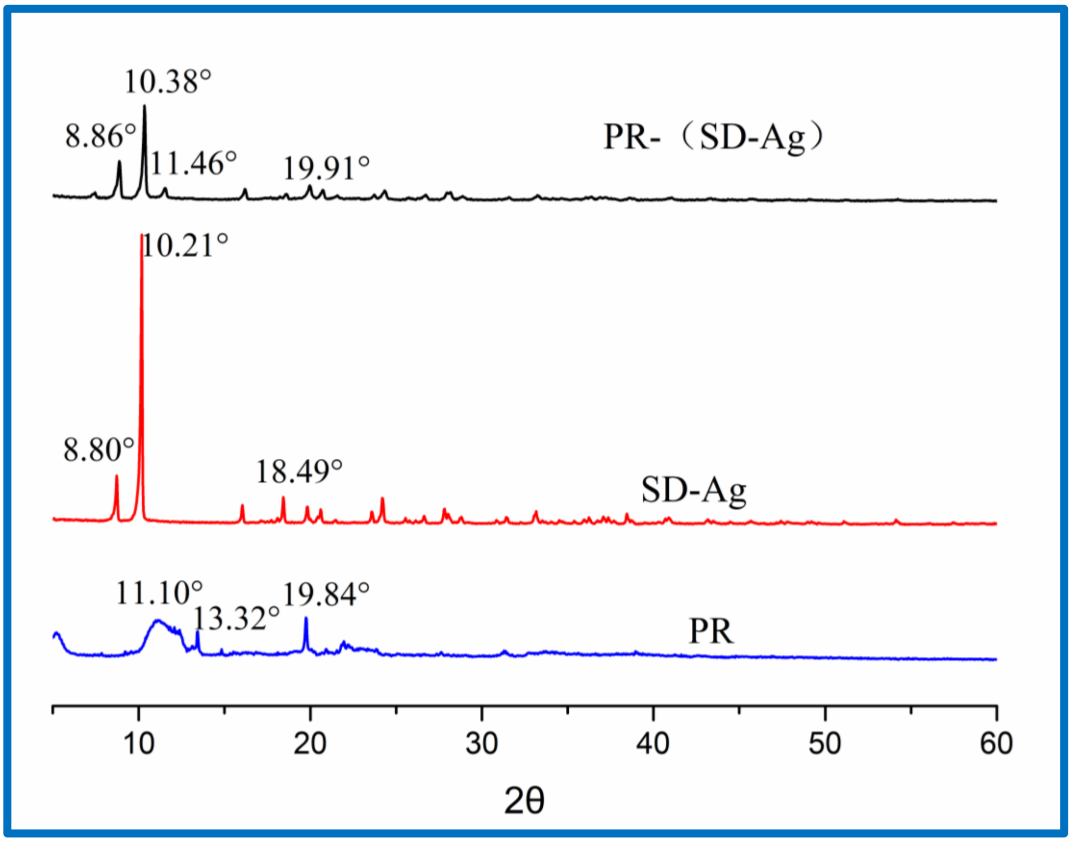

Figure S2 The XRD patterns of PR-(SD-Ag), SD-Ag and PR.

Table S1 Antibacterial rate of $PR_{1\,t}$-（SD-Ag）and $PR_{2\,t}$-（SD-Ag）on E.coli and S.aureus.

**Samples**	**PR/SD-Ag 1:1**		**PR/SD-Ag 1.5:1**		**PR/SD-Ag 2:1**	
	**P(%) E.coli**	**P(%) S.aureus**	**P(%) E.coli**	**P(%) S.aureus**	**P(%) E.coli**	**P(%) S.aureus**
$PR_{1\,1h}$-（SD-Ag）	98.5	98.5	98.3	98.4	98.2	98.1
$PR_{1\,24h}$-（SD-Ag）	98.9	98.8	98.6	98.6	98.4	98.3
$PR_{1\,48h}$-（SD-Ag）	99.1	99.2	98.9	98.9	98.7	98.6
$PR_{2\,1h}$-（SD-Ag）	99.1	98.9	98.8	98.6	98.6	98.4
$PR_{2\,24h}$-（SD-Ag）	99.5	99.1	99.1	98.9	98.9	98.7
$PR_{2\,48h}$-（SD-Ag）	99.9	99.6	99.5	99.3	99.2	99.1

© 2018 by the authors. Submitted for possible open access publication under the terms and conditions of the Creative Commons Attribution (CC BY) license (http://creativecommons.org/licenses/by/4.0/).
